# Advances in Maize Genomics and Their Value for Enhancing Genetic Gains from Breeding

**DOI:** 10.1155/2009/957602

**Published:** 2009-08-12

**Authors:** Yunbi Xu, Debra J. Skinner, Huixia Wu, Natalia Palacios-Rojas, Jose Luis Araus, Jianbing Yan, Shibin Gao, Marilyn L. Warburton, Jonathan H. Crouch

**Affiliations:** ^1^International Maize and Wheat Improvement Center (CIMMYT), Apdo. Postal 6-64, 06600 Mexico, DF, Mexico; ^2^Maize Research Institute, Sichuan Agricultural University, Ya'an, Sichuan 625014, China; ^3^USDA-ARS-CHPRRU, Box 9555, Mississippi State, MS 39762, USA

## Abstract

Maize is an important crop for food, feed, forage, and fuel across tropical and temperate areas of the world. Diversity studies at genetic, molecular, and functional levels have revealed that, tropical maize germplasm, landraces, and wild relatives harbor a significantly wider range of genetic variation. Among all types of markers, SNP markers are increasingly the marker-of-choice for all genomics applications in maize breeding. Genetic mapping has been developed through conventional linkage mapping and more recently through linkage disequilibrium-based association analyses. Maize genome sequencing, initially focused on gene-rich regions, now aims for the availability of complete genome sequence. Conventional insertion mutation-based cloning has been complemented recently by EST- and map-based cloning. Transgenics and nutritional genomics are rapidly advancing fields targeting important agronomic traits including pest resistance and grain quality. Substantial advances have been made in methodologies for genomics-assisted breeding, enhancing progress in yield as well as abiotic and biotic stress resistances. Various genomic databases and informatics tools have been developed, among which MaizeGDB is the most developed and widely used by the maize research community. In the future, more emphasis should be given to the development of tools and strategic germplasm resources for more effective molecular breeding of tropical maize products.

## 1. Introduction

Maize is a widely grown C4 crop with a high rate of photosynthetic activity leading to high grain and biomass yield potential. It is predominantly a cross-pollinating species, a feature that has contributed to its broad morphological variability and geographical adaptability. Depending on the latitude and the climate in which it is grown, maize is classified into three distinct types, tropical, temperate, and subtropical. Maize can also be classified based on: (1) endosperm and kernel constitution; (2) kernel colour: flint, dent, floury, waxy, sweet, and pop corn; (3) maturity; (4) use. Economically, the most important types of maize are grown for grain or fodder and silage production. However, in the tropics, grain is primarily grown for human consumption. FAO predicts that an additional 60 Mt of maize grain will be needed from the annual global harvest by 2030. The demand for maize as an animal feed will continue to grow faster than the demand for its use as a human food, particularly in Asia, where a doubling of production is expected from the present level of 165 Mt to almost 400 Mt in 2030 [1]. 

In addition to being an economically important crop, maize is also a classical genetic model for plant research. It has a number of characteristics that are favorable for an experimental model for crop plants: (i) a multiple-purpose crop with worldwide cultivation which attracts research funding from public and private institutions, (ii) <59 000 [2] and 42 000–56 000 genes [3] with moderate genome size (∼2400 Mb of DNA per haploid nucleus in the B73 inbred, which is approximately six times larger than rice and six times smaller than wheat, although a large proportion of the genome represented by repetitive elements), (iii) outbreeding reproduction system with tolerance of inbreeding, (iv) existence of multiple breeding products (inbreds, hybrids, synthetic cultivars, open-pollinated varieties (OPVs), improved landraces), and (v) wide adaptability including good sources of resistance to environment stresses. 

The objective of this paper is to overview various aspects of maize genomics, including genetic and molecular diversity, genetic mapping and trait tagging, physical mapping and genome sequencing, functional genomics, transgenics, nutritional genomics, genomic databases and tools, and genomics-assisted plant breeding. Throughout the paper we have attempted to synthesize the current status in these areas with a particular reference to implications for impacts on application and molecular breeding. Finally, we provide a brief outlook on future developments in this field and the resultant opportunities that they may provide for the development of new maize products.

## 2. Genetic, Molecular, and Functional Diversity

The maize genome harbors tremendous molecular diversity that mirrors its substantial phenotypic variability. When considering nucleotide polymorphism in genes, two maize lines are on average as diverged from one another as humans are from chimpanzees [4]. Understanding the useful genetic diversity for crop improvement should speed the development of new, more productive and better adapted cultivars.

### 2.1. Molecular Genetic Diversity

Molecular markers have been extensively used in maize genetic diversity studies for: (1) analysis of genotype frequencies for identification of deviations at individual loci [5] and for characterization of molecular variation within or between populations [5–9], (2) construction of “phylogenetic” trees [5, 7, 10–14] and determination of heterotic groups [7, 13, 14], and (3) analysis of correlation between genetic distance and hybrid performance, heterosis (when the hybrid shows vigor superior to its parents), and specific combining ability [11, 15].

Several studies have demonstrated a decline in genetic diversity across the elite temperate maize pool over the past century [16, 17]. This supports earlier conclusions that landraces and wild related species contain untapped sources of biotic and abiotic stress resistance that should provide useful new alleles for future maize improvement [18]. After analyzing over one hundred maize inbred lines and teosinte accessions with 462 SSRs, Vigouroux et al. [19] concluded that many alleles in the progenitor species of maize (teosinte) are not present in maize. Wright et al. [20] compared single nucleotide polymorphism (SNP) diversity between maize and teosinte in 774 genes and concluded that maize accessions had a far lower genetic diversity consistent with products of artificial selection and crop improvement. 

There is a growing awareness that levels and patterns of allelic diversity within specific chromosomal regions provide an important chromosomal context for each locus in that region. “Diversity maps” showing the distribution(s) of allelic diversity across chromosomes and genomes of a variety of organisms suggest that there is an association between chromosomal structural features (such as centromeres and telomeres), and selection in particular well-defined gene pools [21–23]. In addition, diversity analysis of individual genes is shedding new light on crop productivity and evolutionary processes underlying plant domestication [4, 24–26]. Maize is a crop that has high-resolution genetic maps and, therefore, it is an ideal choice for development of diversity maps that could provide new information about the consequences of natural selection, domestication, and polyploidy formation. Correlating variation at the molecular level to phenotypic diversity is an essential prerequisite for future studies of diversity using the large number of available candidate genes. Quantitative trait locus (QTL) information can then be combined with association approaches to select a small number of candidate genes that have a high probability of being directly related to a specific phenotype.

### 2.2. Molecular Marker-Based Core Collections and Allele Mining

Molecular genetic diversity analysis has provided a powerful tool to enable efficient and systematic sampling of the diverse material from breeding programs and germplasm collections. Data from molecular markers have been used to construct core subsets, which maximize the diversity of the original collection in the minimum number of genotypes [27, 28]. The Generation Challenge Program (GCP) has been coordinating the identification of minicomposite germplasm collections for around twenty of the most important staple crops in developing countries. These subset collections are now being intensively characterized at the molecular and phenotypic levels to find new functional diversity for important agronomic traits. Meanwhile, maize is being intensively screened via resequencing and precision phenotyping to test the feasibility of the whole “population” based approach for simply inherited traits. For example, using association mapping and allele mining to identify optimum SNP haplotypes for use in molecular breeding programs [29].

Molecular and functional diversity of the maize genome can be characterized through allele mining, identification of distinct “haplotypes” for different inbred lines, analysis of single feature polymorphisms (SFPs), discovery of nearly identical paralogs (NIPs [30]), and determination of their evolutionary implications. In general, there are two approaches that have been elaborated for allele mining: resequencing and ecotilling [31]. Ecotilling is not being widely used in maize at this time, due to the very high number of sequence differences found between different maize accessions, which confounds the interpretation of the differences in such a complex system. Nevertheless, genomewide genotyping using gene-based markers can be used as the foundation of the resequencing method for allele mining in diverse germplasm. However, this approach suffers from the fundamental challenge of establishing which of the various alleles present is functionally different from the wild type, and identifying which new alleles beneficially influence the target trait. Methods to ascertain allele function include marker-assisted backcrossing (MABC), genetic transformation, transient expression assays, and association analysis using an independent set of germplasm from that used to identify the allele. As more genetic variation is functionally validated, it is hoped that the growing database of comparisons between sequence variation and phenotype will allow bioinformaticians to identify patterns that can form the basis of future predictive models. Meanwhile, the rate limiting factor for the effective use of outputs from allele mining in breeding programs continues to be insufficient information on the relationship between SNP variation and changes in phenotypes that may be useful for breeders. However, resources and tools necessary to perform *in silico* trait targeted selection of the outputs from allele mining are becoming available. Thus, proof-of-concept projects are now being carried out in model organisms, in order to study the relationships between SNP haplotypes and changes in phenotypes. This has already led to the development of predictive tools that can identify those SNPs with a high probability of conferring deleterious phenotypes. However, the next big step in this area is the development of bioinformatics tools to compare sequence variation with protein and functional domain variation or with public databases including associated phenotype data, in order to predict which subselections of SNP haplotype variants have the maximum likelihood of providing beneficial phenotypic variation in the target trait. It is likely that SNPs in promoter and noncoding regions will also be important for predictive phenotype analysis.

The same methodology used in association mapping may also be used for allele mining of the diverse core subsets of maize created from breeder's lines, genebank accessions, and wild relatives. Once a gene of interest is positively identified (via association mapping or any other technique) and the sequence determined, this gene can then be resequenced (entirely or in part) in all the individuals of the subset. Changes in the DNA sequence, corresponding to new alleles of this locus, will be identified in this manner, and individuals carrying the new alleles can be evaluated for the target trait to determine the associated change in phenotype and the value for subsequent use in breeding programs. These alleles may never have been found via simple phenotypic screens, either because it is not possible to grow and measure every plant in a large germplasm collection under all possible environmental conditions, because their effect may be masked in certain genetic backgrounds, or because the effect may be so small that it will not be found unless specifically sought in carefully controlled phenotypic screens.

Sequence validated SFPs between maize inbred lines can be detected by hybridizing RNA or complexity-reduced genomic DNA to an Affymetrix GeneChip expression array. Gore et al. [32] evaluated the efficacy of four different complexity-reduction methods for sensitive SFP detection in maize: cDNA, methyl filtration, high-C_0_ *t* selection, and AFLP. These four methods were applied to three diverse maize inbred lines (B73, Mo17, and CML69) with three replications per line (36 GeneChips). The results indicate that all genome reduction methodologies offer modest power to detect SFPs with the commercially available GeneChip Maize Genome Array.

The cost of sequencing is steadily reducing and sequencing increasing numbers of maize genotypes is becoming increasingly possible. Thus, diversity studies at a functional level based on sequencing can be used to complement (and reveal more fundamental variation) studies using genetic and molecular marker analysis. In a recent example, a massively parallel pyro-sequencing technology commercialized by 454 Life Sciences Corporation was used to sequence the transcriptomes of shoot apical meristems isolated from two inbred lines of maize (B73 and Mo17) using laser capture microdissection (LCM) [33]. Putative SNPs were computationally identified, with over 36 000 putative SNPs detected within 9980 unique B73 genomic anchor sequences. Stringent postprocessing reduced this number to around 7000 putative SNPs. Over 85% (94 of 110) of a sample of these putative SNPs were successfully validated by Sanger sequencing. Based on this validation rate, this pilot experiment conservatively identified more than 4900 valid SNPs within over 2400 maize genes. These results demonstrated that 454-based transcriptome sequencing is a method of choice for the high-throughput acquisition of gene-associated SNPs.

## 3. Genetic Mapping and Gene Tagging

The efficiency and precision of genetic mapping and gene tagging has significantly improved in recent years particularly due to the development of sequence-based markers and array-based genotyping systems. Both private and public sectors have established array-based genotyping systems for SNPs, which makes high-throughput genome-wide genetic mapping possible. A large collaborative effort to determine and define SNP loci in genes has been combined with a highly multiplexed array-based genotyping system for genetic analysis of maize [34, 35]. For example, this can be based on the Illumina GoldenGate assay system which facilitates the simultaneous assay of large numbers of SNP loci (e.g., 1536 as currently available) in an arrayed format.

### 3.1. Linkage Mapping

Genetic mapping in maize was first carried out using morphological markers generating a genetic map consisting of 62 morphological trait loci [36]. The first generation of molecular marker maps in maize was constructed using restriction fragment length polymorphisms (RFLP) [37, 38], which were later saturated with simple sequence repeat (SSR) and other types of PCR-based markers [39]. Most recently, linkage mapping is being raised to a new level as maps are being developed with large numbers of SNP markers and/or candidate gene-based markers.

Linkage mapping, mainly using RFLPs and SSRs, has been carried out in maize by numerous laboratories since the 1980s and large amounts of data are now available in the MaizeGDB database (http://www.maizegdb.org). Several types of mapping population have been used, including F_2_ [37, 40], immortalized F_2_ [41, 42], and populations of recombinant inbred lines (RILs) [38, 43, 44]. These mapping efforts have involved up to 214 individuals screened with 92 to 1736 markers. Composite maps have also been constructed from multiple crosses [45]. In order to improve the resolution and extend the total map distance, the Maize Mapping Project (MMP) developed RILs through several generations of intermating an F_2_ population derived from the single cross of the inbred lines B73 and Mo17. As a result, the resolution of the genetic map was improved significantly, consisting of about 1000 RFLP and 1000 SSR markers (see [39]; MaizeGDB, www.maizegdb.org). A second panel of intermated RILs (IRILs) developed from F2 × F252 was used for linkage mapping of 1454 maize candidate genes [46], which created a higher map resolution with more cDNA loci mapped than when using nonintermated RILs. The maize sequencing project (using inbred line B73) and the constant progress in maize functional genomics are providing new genes and functional genomic DNA sequence information that are increasingly being integrated into the maize genetic map [47]. A total of 25 908 markers have now been integrated into the fingerprinted BAC contig (FPC) map [48]. This includes 1902 genetically mapped markers (SSRs, RFLPs, SNPs, and InDels) and 24 006 sequence-based markers (ESTs, BAC ends, and 40-bp overlapping oligonucleotide overgo probes) [49]. Compared to other types of molecular markers, SNP markers have several advantages, including high abundance and even distribution through the genome. In addition, SNP markers provide highly reproducible codominant information, and there is an increasing range of cost effective high-throughput SNP genotyping systems. It would be expected that the second generation of molecular markers such as SSRs will soon be replaced by third generation SNP markers in genomics research and genomics-assisted breeding. At the International Maize and Wheat Improvement Center (CIMMYT), over 2000 SNP markers that were developed for SNP chip-based genotyping through collaboration with Cornell University and Illumina Inc are being mapped using three RIL populations.

### 3.2. Gene/QTL Mapping

Molecular marker-facilitated mapping of genes underlying specific traits in maize was first reported in the late 1980s by Stuber et al. [50] followed by Edwards et al. [51] and Stuber et al. [52]. Since then, more than 2000 QTL related to various traits of agronomic importance in maize, including yield, yield components, plant morphology and physiology, and biotic and abiotic stress responses have been reported (www.maizeGDB.org). Two examples which address special issues related to typical quantitative variation should be mentioned here. In one study, two elite inbred lines were crossed to create a 1000-individual mapping population which was evaluated in 19 environments for grain yield, grain moisture and plant height [53]. In the second example, the high and low oil and protein content lines derived from 70 generations of long-term selection were crossed, intermated, and mapped [54]. Both studies reported numerous QTL of very small effect, supporting the concept that quantitative variation is the product of numerous minor genes. This hypothesis is also supported by long-term large-scale QTL studies on drought tolerance in maize that have found no “major effect QTL” [55]. However, as highlighted by Buckler et al. [4], in all these cases, the parental genotypes of the mapping populations probably tapped only a small proportion of the functional variation available in the maize genepool. Plus some important QTL for complex traits may be fixed in the advanced breeding material used as parental genotypes in some of these studies.

Most reported QTL have been identified using bi-parental mapping populations. This approach suffers from the problem that the estimated position of the QTL can vary significantly if different genetic backgrounds are used. This is particularly troublesome for more complex traits such as yield, yield components and abiotic stress tolerance. In addition, these traits have low heritability and high genotype-by-environment interaction (GEI). Hence, these QTL results may only be applicable to breeding populations closely related to the mapping population and that are targeted for same environment conditions as used for the evaluation of the mapping population. Thus, it is important to provide more generally applicable information for the development of marker-assisted selection (MAS) systems and to expand the utilization of QTL results in a range of breeding populations. This may be achieved by surveying the overlapping regions or colocalization of QTL for the same trait across different mapping populations and diverse growing conditions using a core set of markers to generate a consensus map. Bioinformatic tools have been developed to facilitate integration, comparison, and collective analysis of such data. For example, the Comparative Map and Trait Viewer (CMTV) was developed to integrate data collected by CIMMYT in maize drought tolerance research with data from public sources [56]. In addition, QTL for grain yield, kernel weight, abiotic response [57], and disease [58] have been integrated into a consensus map based on data available at the web-based MaizeGDB database. A comprehensive review of all QTL studies in maize is beyond the scope of this paper. However, Tuberosa and Salvi [59] have summarized the main results from the large number of studies that have described QTL for grain yield and other agronomic traits in maize.

Two methods that can save time and money when attempting to identify markers that tag QTL are bulked segregant analysis (BSA) and selective genotyping (reviewed in [60]), where only selected individuals representing the two phenotypic extremes of the target trait are genotyped. BSA is based on bulking the DNA of the selected plants while selective genotyping focuses on individual plants. Selective genotyping can be used to replace entire population genotyping if a sufficient population size is used. This has the advantage of focusing on the individuals that contribute the most useful data to the analysis while then being able to increase the number of markers applied [61, 62]. These two methods are particularly useful for identification of large-effect or major QTL. BSA has been successfully applied to detect major QTL in maize for yield and leaf abscisic acid (ABA) concentration under drought conditions [63], which were not easy to detect with traditional mapping populations. To develop maize molecular breeding systems, CIMMYT scientists are now using large-scale selective genotyping to test whether elite alleles for drought tolerance, disease resistance and grain quality traits can be identified among large numbers of carefully chosen breeding lines that are genotyped with a large number of SNP markers. 

QTL mapping has also been used to study the domestication process for cultivated maize. Domesticated maize and its wild progenitor, teosinte, differ dramatically in their overall plant architecture and the morphology of their female inflorescences. Five major QTL have been identified that differentiate maize from teosinte [64]. Two QTL conferring major morphological differences were defined as single Mendelian loci: *teosinte branched1* (*tb1*) [24, 65, 66] and *teosinte glume architecture1* (*tga1*) [67, 68]. The allele *tb1* suppresses lateral branching (leading to apical dominance) while *tga1* affects the hardness of the seed coat (hard casing that envelops the seed in its ancestor teosinte), and both genes were involved in the evolution of cultivated maize from teosinte. More recently, additional key loci controlling the differences between maize and teosinte have been identified through QTL analyses of maize-teosinte intercross progeny using a total of 1723 progeny genotyped with more than 300 SNP, SSR, and candidate gene markers and phenotyped for 22 morphological traits [69]. In another report, the regions in the maize genome that affect tassel branch number were identified [70].

### 3.3. Association or LD Mapping

Association mapping or linkage disequilibrium (LD) mapping has become an increasingly important tool for gene mapping in maize [71–80]. LD often declines to within 1 to 1.5 Kb in maize landraces and a broad range of tropical and temperate inbred lines [81, 82], but with less rapid decay in elite breeding materials [83]. Association mapping in plants can be based on candidate genes or whole genome scanning. The latter has become increasingly applicable in maize due to the recent development of large numbers of SNP markers.

Comprehensive association mapping studies are being carried out in the Buckler group at Cornell University (http://www.maizegenetics.net/), where sequence variation in thousands of genes with important functions is being studied across a diverse panel of 288 maize genotypes. CIMMYT is currently applying association mapping techniques in order to identify markers for provitamin A accumulation in colored maize kernels. CIMMYT is also using these approaches for more complex traits such as drought tolerance, using drought tolerance related candidate genes and phenotyping over 300 diverse maize genotypes in multiple locations over two years. 

To facilitate genome-wide association mapping of both qualitative and quantitative traits, an integrated mapping strategy, Nested Association Mapping (NAM), was designed consisting of 25 maize populations, each of which has 200 RILs derived from crossing one of 25 diverse inbred lines to a common inbred [84]. With a dense coverage (2.6 cM) of common-parent-specific (CPS) markers, the genome information for 5000 RILs can be inferred based on the parental genome information. Essentially, the linkage information was captured by the CPS markers and the LD information among loci residing between CPS markers was then projected to RILs based on parental information, ultimately allowing for genome-wide high-resolution mapping. The power of this approach using 5000 RILs allows 30% to 79% of the simulated QTL to be precisely identified [84, 85]. Preliminary analysis of flowering time has led to the identification of 50 QTL significant beyond the LOD 4 threshold. Although there was a 40-day variation in flowering time between the tested lines, no allele with more than 3-day effect on flowering time was observed and the vast majority of the allelic effects provided just 0.5–1.0 day changes in flowering time. Next steps include QTL identification, examination of the recombination events in specific populations, and association analysis of SNP datasets. This will lead to the identification of single genes or candidate genes underlying key QTL controlling the target traits (Ed Buckler, personal communications). Combining the complete genome sequence with NAM approach will greatly facilitate the dissection of complex traits in many species able to apply this strategy. At CIMMYT, a strategy has been developed to integrate linkage- and LD-based mapping. This is being used for mapping drought tolerance using two 1536-SNP chips, one of which was developed from candidate genes related to drought tolerance [86]. Compared to linkage-based mapping, the advantages of LD-based mapping are: no need for generation of mapping populations; high mapping resolution; mapping of multiple traits using the same set of germplasm. However, the power of association mapping is variable. Although whole genome scan mapping of traits has been validated in maize, in general, we do not have a detailed understanding of the power of this approach, given the highly variable LD, noisy phenotypic data and complex population structure [87].

### 3.4. Validation of Marker-Trait Associations

A wide range of approaches to validation of marker-trait associations has been reported, each with their own profile of advantages and disadvantages. The availability of thousands of SNP markers, rather than hundreds of SSR markers, makes it practical to validate marker-trait associations through high-precision genotyping of an independent set of parental lines and breeding populations. Marker validation can also be carried out by simultaneously mapping multiple populations or by selective genotyping of phenotypic extremes from multiple relevant breeding populations. A similar level of validation may be achieved when applying association mapping with a large number of diverse inbred lines. Greater efficiency in the validation process can be achieved through pooled DNA analysis provided that markers have been selected and optimized for this approach. However, validation requirements may be minimized when dealing with large-effect QTL [88]. Alternatively, the “mapping-as-you-go” approach provides the opportunity to validate and/or refine the marker-trait associations at every generation [89]. It is likely that approaches based on the use of breeding materials for mapping will become increasingly popular due to the overall time and cost efficiency. At the same time, it is likely that the utilization of haplotype-based selection rather than single-marker-based selection will become increasingly common in view of the increased selective power that it provides, particularly for complex traits and/or multiple trait selection.

An alternative strategy for confirming candidate QTL is the use of near-isogenic lines (NILs). Reducing much of the “noise” caused by the effects of genetic background, NILs offer much more accurate estimates of QTL effects than RILs, particularly if multiple QTL are segregating in the population. However, the power to detect an individual QTL may be greater in RILs than NILs [90, 91]. For example, a major L-ABA (leaf-abscisic acid concentration) QTL in bin 2.04 affecting root traits and relative water content was further confirmed using NILs [63]. Grain yield [92] and flowering time [91] traits have also been mapped using this method.

## 4. Whole Genome Sequencing

Two large-scale efforts in the USA were launched during the early 2000's to sequence the maize genome. The Sequencing the Maize Genome Project (STMG) was a collaboration between the Plant Genome Initiative at Rutgers University (J. Messing), and the Arizona Genomics Institute and Computational Laboratory (R. Wing and C. Soderlund). Working from the high-resolution FPC genome sequencing began by producing BAC-end sequences (BES, also known as Sequence Tag Connectors [STC]) [2]. These sequences help to order the BAC's as well as provide novel sequence and a repeat sequence database. Shotgun-sequenced BACs provide valuable new sequence and also important data on genome structure [3]. 

Given the structure of the maize genome, strategies for targeting gene rich regions have been the focus of the second major maize sequencing initiative. The Consortium for Maize Genomics (CMG), consisting of the Donald Danforth Plant Science Center, The Institute for Genomic Research (TIGR), Purdue University and Orion Genomics, has used selective techniques for enriching for genes. Two strategies, methyl-filtration and high-*C*_0_ *t* selection, were used to enrich for gene rich regions [93, 94]. Methyl filtration is based on the finding that the genic regions of maize are not methylated, and can be selected accordingly. High-*C*_0_ *t* selection follows the rate of rehybridization of genomic fragments following denaturation, with repetitive sequences reannealing quicker than low copy number genic sequences. Both strategies have been shown to be very effective, capturing 95% of genes in test BACs, and are likely to be more efficient than random whole genome shotgun sequencing alone [95, 96]. Assembled *Zea mays* sequences from these techniques can be viewed at TIGR (http://maize.tigr.org/release4.0/assembly.shtml). In addition, the MAGIdb contains Maize Assembled Genomic Islands (MAGIs) using almost four billion genome survey sequences generated through the CMG which can be viewed and used to BLAST against other sequences (see [97]; http://magi.plantgenomics.iastate.edu/) with basic local alignment search tool (BLAST).

In addition to these efforts, other methylation based methods developed for maize will help to extend genic “islands” and also order these islands relative to each other. Hypomethylated partial restriction (HMPR) libraries generated by methylation sensitive restriction enzymes can be larger than the methyl filtration clones as they are tolerant of internal methylation sites [98]. Therefore, they can cover the gene island and extend the available sequence for any particular gene. Another approach, methylation spanning linker libraries (MSLLs) also uses methylation sensitive enzymes but selects for large fragments, where the ends are anchored in neighboring genes, indicating linkage, orientation, and distance between genes [99]. 

A full-scale program to sequence the entire maize genome was initiated in 2005 through the NSF-funded Maize Genome Project as a collaboration between the Washington University Genome Sequencing Center, the Arizona Genomics Institute (AGI), Iowa State University, and Cold Spring Harbor Laboratory, aiming to sequence the maize genespace of the cultivar B73 to a finished quality using a BAC-based approach (NSF award DBI-0527192). The strategy is to utilize a minimal tiling path of approximately 19,000 mapped BAC clones to focus on producing high-quality sequence coverage of all identifiable gene-containing regions of the maize genome. These regions will be ordered, oriented and, along with all of the intergenic sequences, anchored to the Agarose FPC physical map and the IBM genetic maps of the maize genome (maizesequence.org). Development of maize physical map of the B73 inbred is essential for the use of the sequence information. In February, 2008, at the 50th Maize Genetic Meeting, the Maize Sequencing Consortium announced the draft sequence of the maize genome. Since then, extensive work has been done to finish the remaining clones, improve the physical map, anchor the sequence to the genetic map, build an AGP (A Golden Path) to generate maize pseudomolecules, and to annotate the genome. On March 20, 2009, AGI released an updated maize integrated genetic and physical map, consisting of 440 contigs. 

A recent report (ScienceDaily, June 28, 2008) indicates that a major maize sequencing project is being carried out at the Center for Research and Advanced Studies of the National Polytechnic Institute (CINVESTAV, Mexico), using bulked plants from the Mexican popcorn landrace Palomero. This maize accession has 22% less DNA and is phylogenetically closer to teosinte than B73. The project has been focusing on important gene rich regions (http://www.niherst.gov.tt/s-and-t/s-and-t-news/; Dr. Alfredo Herrera Estrella, CINVESTAV, personal communications). Structural and functional analysis of this genome reveals a large number of unreported sequences, suggesting that the ancient landraces contain a large pool of unexplored genetic diversity that could be useful in new crop generation as well as the study of the evolution and domestication of maize and other cereals (http://abstracts.aspb.org/pb2008/public/S02/S024.html).

With next-generation DNA sequencing technology [100, 101], we may be able to sequence any maize genotype of interest. A new generation of high-throughput sequencing technologies promises to transform the scientific enterprise, potentially supplanting array-based technologies and opening up many new possibilities [102]. Significantly enhanced sequencing throughput will allow us to uncover the huge diversity of novel genes that are currently inaccessible, to better integrate biological information for a complete picture of many traits at an individual level and to move to advances that we cannot yet imagine.

## 5. Functional Genomics

Functional genomics seeks to determine gene functions and interactions using genomic scale data. An important part of new gene discovery through EST and genome sequencing is the annotation of those genes to assign putative functions. In the absence of empirical data for a particular gene in the appropriate target organism, gene annotation software can predict a function using data from rice, *Arabidopsis* and other organisms based on similarities for intron/exon sequence and structure plus likely protein domains (www.maizesequence.org). Users can BLAST their own maize sequences against the cereal and other species databases to generate predicted function information. In forward and reverse genetics approaches, maize is characterized by excellent mutagenesis resources in the form of well-studied transposon systems, and new techniques for induced mutations are also being applied [103]. The completion of the maize genome sequence provides the most essential resource to move easily from gene to mutant phenotype and back. There are several methods for experimentally determining gene function. Only three important fields will be discussed.

### 5.1. Gene Cloning

Major challenges for gene discovery include: (1) the large size of the maize genome; (2) variation in genome size and gene order; (3) the high incidence of multicopy genes; and (4) transposons and other repetitive sequences make up a large portion of the genome. Traditionally, gene discovery in maize has employed transposon tagging, EST searches and comparative genomics, and due to the increase in genomic resources, positional cloning is increasingly being used for both qualitative and quantitative traits. 

Positional cloning in *Arabidopsis* and rice has been very successful because of their small genomes and the availability of the complete genome sequence. Until recently, positional cloning in maize was considered nearly impossible because of the vast amounts of repetitive DNA. However, now with the physical maps for maize, the large numbers of available markers, and critically, conservation of synteny across the cereal genomes, it is feasible to consider a chromosome walk in comparable or less time than cloning by transposon tagging [104]. The basic steps are the development of a large segregating population, of at least 1000 individuals, and initial mapping using a subset of the population with around two markers per chromosome arms to generate an approximate map position of the gene of interest. The remainder of the population is then genotyped with the flanking markers to identify recombinants from the whole population that are suitable for the fine mapping process. Additional markers are then screened across these recombinants, and where markers from maize are not available, syntenic rice or sorghum markers can be used. These methods should allow determining the physical position of the target gene at the level of an individual BAC contig which equates to an average distance of 1 cM [104]. However, this depends on highly precise phenotyping, and is more rapid for qualitative traits whose phenotypes are easily determined. To assist the process, outputs from the maize genome project (including overgos, anchoring of ESTs, and BES) and the fully sequenced genome of rice can be used to identify candidate genes. These genes can then be sequenced to look for the mutation and in the process could provide additional markers to narrow down the location of the target gene. As more genomics resources become available, including the genome sequence of maize and its relatives, positional cloning of quantitative traits will be most limited by the need for precision phenotyping of the trait under study.

An important and surprising finding from comparing sequence data from Mo17 and B73 is that on average 50% of the sequence at a genomic locus can differ between these two inbred lines [105]. This might be expected to significantly interfere with the positional cloning process. However, the majority of sequence differences between inbreds are caused by insertion or deletion of partial genes or pseudogenes carried by *Heliotron * transposons. The genes that are collinear between inbreds are usually collinear with rice, meaning that if the rice genome is used as a reference for positional cloning it is unlikely to be affected by the differences between maize inbreds. Where the genomic differences between inbreds are significant, more work will be needed to find useful polymorphisms, but given the extensive diversity between maize lines, these polymorphisms are likely to be abundant. On the other hand, however, it is not uncommon to see genes that are contiguous in rice being split across two regions in maize [106, 107]—this can make cloning difficult. The first genes identified by positional cloning in maize were QTL as these were more difficult to target with transposons. However, this type of trait presents a serious challenge due to the difficulty of accurately screening the phenotype and the need to work with large population sizes. A maize domestication locus, *teosinte glume architecture* (*tga1*), which encodes a transcriptional regulator, was the first maize gene positionally cloned, using a population of over 3000 individuals [108]. A QTL for flowering time was localized to a conserved 2 kb noncoding region using a combination of positional cloning, comparative genomics, and association (linkage disequilibrium) mapping. The noncoding region was shown to act as an enhancer of a distant flowering gene [77].

Positional cloning was recently used to identify an RNA polymerase encoding gene (*rmr6*) which influences paramutation and maize development [109]. In this case, the predicted maize genes within the mapped interval were absent from the syntenic interval in rice, but four of these predicted genes formed a single large gene which was homologous to an Arabidopsis polymerase gene. Several genes that regulate inflorescence architecture in maize have also been cloned. The gene responsible for the mutated phenotype of a highly branched tassel and a branched ear, *ramosa 1* (*ra1*), was cloned by transposon tagging [110]. Two other maize inflorescence genes have been cloned using the map position of the mutation in combination with prior candidate gene information. The *barren stalk 1* (*ba1*) mutants lack tassel branches and spikelets and are missing ears. The positional cloning of *lax panicle* in rice [111] provided a candidate gene for *ba1*. In a second example, a maize *clavata 1* (*clv1*) ortholog was mapped to chromosome 5 in the same region as *thick tassel dwarf 1* (*td1*). The phenotype of *td1 * mimics that of *Arabidopsis clv* mutants, which have larger inflorescence meristems and more floral organs. Proof that *td1* was the *clv1* ortholog came from analysis of a large number of Mu-induced alleles [112]. In addition, *ra2*, *ra3*, *tasselseed4* (*ts4*), and *sparse inflorescence1* have also been cloned using the positional approach described above [113–115]. Cloning of *indeterminate gametophyte 1 * used a combination of positional cloning and transposon insertion to validate candidate genes [116]. 

Silage maize is an important source of forage for dairy cattle due to its high energy content and good digestibility. Brown midrib (*bm*) mutants in maize have an increased digestibility but inferior agronomic performance [117]. Two of the four *bm* genes (*bm1* and *bm3*) have been shown to be involved in monolignol biosynthesis [118]. These two and additional lignin biosynthesis genes have been isolated based on sequence homology with rice. Candidate genes putatively affecting forage quality have been identified by expression profiling using isogenic *bm* lines, and associations detected between a polymorphism at the COMT (caffeic acid O-methyl transferase) locus and DNDF (digestible neutral detergent fiber) in a collection of maize inbred lines [119, 120]. Conserved domains or motifs shared by across known resistance genes have been extensively exploited to identify unknown resistance gene analogs (RGA). In an attempt to isolate all potential RGA based on these domains, three approaches were adopted by Xiao et al. [121], including the modified AFLP (amplified fragment length polymorphism), modified RACE (rapid amplification of cDNA ends), and data-mining methods. In response to herbivorous insects, plants synthesize and release volatile chemical signals that will attract the natural enemies of the herbivore to defend themselves. A recent report described the findings that the function of the maize *Hm1* resistance gene may be conserved in other grasses to protect these plants against the lethal fungal necrotrophic pathogen *Cochliobolus carbonum* [122]. Lin et al. [123] reported the isolation and characterization of the maize monoterpene synthase gene *tps26* that is an ortholog of *stc1*, a gene induced in response to the attack of beet army worm larvae. Maize orthologs of the tomato *pto-interacting* (*pti*) protein have been identified through homology and library screening [124]. The tomato gene functions in signal transduction of the defense response to *Pseudomonas syringae, * while at least one of the four *ZmPti1* genes functions in pollen signaling. Examples of functionally characterized genes in maize include *Dwarf8* that encodes a gibberellin response modulator from which functional markers can be developed for plant height and flowering time. For example, nine sequence motifs in the *Dwarf8* gene of maize were shown to be associated with variation in flowering time, and one 6 bp deletion accounted for 7–11 days difference in flowering time between inbreds [71]. However, *Dwarf8* is a pleotropic gene (also affecting plant height) and thus there is a need to identify functional markers from additional flowering time genes for use in breeding applications. Recently, a streamlined PCR-based cloning strategy and massively parallel sequencing are used to clone and characterize the expression of a maize COBRA-like gene required for cell wall expansion in root hairs [125]. A list of additional characterized maize genes is maintained at MaizeGDB (http://www.maizegdb.org/newgenes.php).

### 5.2. Transposon Tagging and TILLING

The wealth of active transposable elements residing in the maize genome plays an important role in functional genomics. In addition to serving as molecular tags for mutated genes, these transposons tend to knock out genes into which they insert. Mutant libraries constructed using transposon tagging, T-DNA insertion, and chemical and physical mutagenesis provide materials that can be screened for base changes in any genes by reverse genetics methods such as Targeting Induced Limited Lesions IN Genomes (TILLINGs). 

Large populations of maize plants containing highly active Mutator transposons have been created to saturate the genome with insertional mutations, and these form the basis for different transposon tagging resources (reviewed in [103]). May et al. [126] described an efficient system for site-selected transposon mutagenesis in maize. A total of 43 776 F_1_ plants were generated by using Robertson's Mutator (Mu) pollen parents and self-pollinated to establish a library of transposon-mutagenized seed. The frequency of new seed mutants was between 10^−4^ and 10^−5^ per F_1_ plant. As a service to the maize community, insertions in genes of interest from this library can be selected by using the PCR. A maize-targeted mutagenesis database was established for storing pedigree, knockout, sequence, phenotype, and other information. 

McCarty et al. [127] implemented a novel strategy for harnessing the power of high-copy transposons for functional analysis of the maize genome, and reported behavioral features of the Mutator system in a uniform inbred background. The established unique UniformMu population and database facilitate high-throughput molecular analysis of Mu-tagged mutants and gene knockouts. The Mu populations differed markedly in the occurrence of Mu insertion hotspots and the frequency of suppressible mutations. The public database (http://uniformmu.org; http://endosperm.info) contains pedigree and phenotypic data for over 2000 independent seed mutants selected from a population of 31 548 F_2_ lines and integrated with analyses of 34 255 MuTAIL sequences. 

Chemical and radiation-induced mutations have been widely used for random mutagenesis in plants, resulting in a broader spectrum of mutation alleles that occurs randomly in the genome. A number of chemicals have been used to generate large mutant collections, many of which are described in MaizeGDB and available for study. Chemical agents generate a broader range of DNA alternations; these are predominantly single base-pair substitution, but also induce small insertions and deletions. Ethyl methane sulfonate (EMS), a base-alkylating agent that generates point mutations (of which the vast majority are G/C-A/T transitions, often leading to the creation of stop codons/nonsense mutations), has been used most commonly because of its ease of use and the diversity of potential mutants. 

Strategies have been developed so that subtle changes like point mutation generated by EMS can be detected easily. For efficient adaptation of chemical induced mutagenesis for reverse genetics in *Arabidopsis* and other plants, McCallum et al. [128] developed the TILLING screening system, which allows a point mutation to be identified at a large-scale. In the basic TILLING method, seeds are mutagenized by treatment with EMS. These mutagenized lines serve as a general forward genetic resource. EcoTILLING [31], as a variant of TILLING, examines natural genetic variation in populations and has been successfully utilized in animals and plants to discover SNPs. The protocols developed for TILLING have been adapted in EcoTILLING for discovery of natural nucleotide variation linked to important phenotypic traits. Till et al. [129] reviewed the current TILLING and EcoTILLING technologies and discussed the process that has been made in applying these methods to many different plant species. In maize, the Maize TILLING Project (MTP) started doing TILLING screens in 2005 using mutant populations created in different laboratories (http://genome.purdue.edu/maizetilling/). Weil and Monde [130] provided a detailed protocol for maize TILLING including TILLING mutagenesis, tissue collection, DNA preparation and 2D pooling, and detailed TILLING workflows. As the maize genome is completely sequenced, advances in reverse genetics technologies including TILLING, EcoTILLING, and massively parallel DNA resequencing provide excellent methods for identifying mutations in a wide variety of traits and biological processes [131].

### 5.3. Transcription Profiling

To provide comprehensive, low-cost, and public sector long-oligonucleotide (around 70 mers) microarrays for gene expression analysis in maize, the first generation of low cost oligonucleotide spotted microarrays was developed for the maize community [132]. A total of 57 452 70 mer oligonucleotides were designed to represent 25 969 ESTs assemblies, 20 206 singleton EST (detected only in a single cDNA library), 9707 assembled maize sequences, 804 nonredundant repeat elements, 467 organelles, 288 maize community favorites and 11 transgenes. Replicated baseline expression profiles have been generated for 18 tissues and deposited in Zeamage as have the results of other expression studies (www.maizearray.org). The GeneChip Maize Genome Array manufactured by Affymetrix (www.affymetrix.com) is a commercial alternative to the public 70 mer array described above. This array contains 17 555 probe sets, which interrogate 14 850 maize transcripts representing 13 339 maize genes. These arrays have 25 mer probes, with 15 different probes designed from the 3′ end of each gene. A recent advance includes whole-genome transcript profiling with a 100 K Maize Affymetrix GeneChip Array, which contains 100 000 probe sets to detect transcripts from *Zea mays*.

Transcript abundance levels differing between the parental genotypes of a mapping population and segregating among the progeny can be mapped and characterized as quantitative traits [133]. Microarrays have been used to determine the gene expression levels and identify genomic regions (gene expression QTL, or eQTL) associated with transcript variation in coregulated genes. The eQTL mapping involves expression profiling as measured by mRNA transcript abundance for a large number of genes which are each treated as a quantitative phenotype likely to be conveyed by multiple genes and influenced by environmental factors. These expressional profiles then constitute a marker-based fingerprint of each individual in a segregating population and can be subjected to conventional QTL analysis [134] albeit interpreted in the spatially and temporally specific context in which the data were collected. 

As one of several combined approaches, gene expression profiling has been used to study the molecular basis of heterosis in maize. In a recent eQTL report, all possible modes of gene action, including additivity, high- and low-parent dominance, underdominance, and overdominance, were observed in a comparison of global gene expression in a maize F_1_ hybrid compared with its inbred parents, B73 and Mo17 [135]. Over one thousand genes were identified as being significantly differentially expressed between the three genotypes. In a second report, microarray analysis of gene expression patterns in immature ears, seedlings, and embryo tissues from the inbreds B73 and Mo17 identified numerous genes with variable expression. The reciprocal F_1_ hybrid lines did not display maternal or paternal effects on gene expression levels. The results suggest that cis-transcriptional variation between B73 and Mo17 led to additive expression patterns in the F_1_ hybrid [136]. This group has also reported other properties of gene expression in some maize hybrids [137–139]. In a third report, a wide-scale survey of transcriptional heterosis in maize immature ears has been carried out using B73 and H99 inbred lines and the resultant F_1_ using cDNA microarray technology and real-time PCR (RT-PCR) [140]. Genes expressed at a significantly different level between parental genotypes and the F_1_ hybrid were identified. Both dominance and overdominance components were reported to be involved in nonadditive gene expression variation in the studied ear developmental stage, encompassing a wide variety of biological processes. Other examples of nonadditive gene expression have been described in a root transcriptome study, which identified a gene that was consistently expressed above the midparental value for different parent and hybrid combinations that may function in heterosis [141]. In addition, massively parallel signature sequencing (MPSS) [142], a deep sequencing-based mRNA profiling technology, was used to study allele specific expression. This enabled a genome-wide evaluation of cis- and transeffects on allelic expression in six meristem stages of the maize hybrid [143].

Gene expression profiling has been widely used in the study of abiotic stress tolerance (e.g., [144] as reviewed in [145–147]). In maize, flowering is the developmental stage most vulnerable to abiotic stress leading to significant yield loss associated with aberrant floral development and impaired ear and kernel growth. Genes within the starch biosynthetic pathway are collectively downregulated under stress leading to reduction in starch content. Comparative profiling of the sense and antisense transcriptome revealed that transciptomes of the three lines tested (A619, ND101/W23 and W23) displayed remarkable similarities across four tissues (leaves, 1 mm anthers, 1.5 mm anthers, pollen) despite high levels of polymorphism and structural differences between the inbred lines [148]. Transcriptome analysis of the low-phosphorus responses in roots and shoots of a phosphorus-efficient maize line identified alterations of several metabolical and physiological processes [149]. Maize seedlings were surveyed for transcription changes under six abiotic stresses, including the agronomically relevant treatments cold and dessication [150]. Overlapping expression profiles and coordinate expression indicated genes relevant to stress resistance, and such datasets form an excellent resource for identifying candidate genes through positional cloning or association mapping. 

Transcription profiling has increasingly become an important genomics tool for gene functional analysis. Research results that have been published so far revealed great variation in gene expression for a large number of genes. However, further functional analyses are needed to understand how these genes contribute individually and together to a specific differential phenotype. As numerous QTL have been located in chromosomal regions using molecular markers and the interaction effects between these have been studied, transcription profiling may follow a similar process beginning with discovery of large numbers of eQTL and then focusing on ones with large effect for more detailed functional analyses and analysis of interaction effects. Before their function can be concluded, it needs to be confirmed that all putative eQTL have a specific influence on the target trait.

## 6. Transgenics

Genetic transformation in major cereal crops has become a powerful research tool for gene validation, as well as enabling the introduction of novel genes directly into breeding pool and thus accelerating or complementing conventional breeding efforts. In maize genetic transformation has been extensively used in crop improvement, particularly for the development of new commercial pest and herbicide resistant cultivars but more recently also including more complex traits such as grain quality and drought tolerance.

### 6.1. Transformation Methodology

The past ten years have witnessed extensive efforts toward the development of an efficient *Agrobacterium*-mediated transformation system for maize with particular emphasis on increasing the efficiency and extending the range of amenable genotypes [151]. Although the biolistic approach (physically shooting DNA into cells) has revolutionized the genetic transformation field for major crop species, it is usually associated with high copy numbers, transgene silencing and rearrangement. *Agrobaterium*-mediated transformation approach (biologically facilitated entry of DNA) is believed to generate a high proportion of independent events with single or low transgene copy numbers which is expected to favor consistent transgene expression in progeny generations [152, 153]. However, since *A. tumefaciens* naturally infects only dicotyledonous plants, monocotyledonous plants remained inaccessible for many years despite enormous efforts worldwide. The breakthrough was made in 1994 when Hiei et al. [154] reported using *A. tumefaciens* to transform rice. Two years later, Ishida et al. [155] reported that the successful *Agrobacterium*-mediated transformation of the maize inbred line A188 and its hybrid using superbinary vectors (cloning vectors, able to replicate in both *E. coli* and *A. tumefaciens*. A superbinary vector carries additional virulence genes from a Ti plasmid), and this was confirmed by Negrotto et al. [156] and Zhao et al. [157]. Subsequently, it was demonstrated that maize could also be transformed using *A. tumefaciens* carrying an ordinary binary vector [158, 159]. For achieving good transformation efficiency, advances have been made through selection of basal media, modifying medium components, optimizing different culture stages, and adding *Agrobacterium* growth-inhibiting agents such as silver nitrate [157, 159–163]. Yang et al. [164] found that the purine and pyrimidine biosynthesis inhibitors, mizoribine, azaserine, and acivicin could induce higher transformation efficiencies when appropriate concentrations were added before or during inoculation with *Agrobacterium*. It is believed that this is achieved by inhibiting key enzymes in the *de novo* purine biosythesis pathway in *Agrobacterium* cells thus improving the competence of plant cells [165]. In addition, a replication-associated protein (RepA) was used to stimulate cell division and callus growth leading to higher transformation efficiency [166]. More recent developments in transformation technology also include development of novel plasmids and T-DNA binary vectors that incorporate a modified and more useful form of the superpromoter [167] and construction of engineered minichromosomes by modifying natural A and supernumerary B chromosomes [168].

Only a limited number of proprietary [166] or public inbred lines [169–171] and various recalcitrant inbred lines crossed to A188 [161, 172] have been transformed using *A. tumefaciens*-mediated transformation. Zhao et al. [157] reported using Hi-II which contains A188 and B73 genetic backgrounds, for achieving high-transformation efficiencies up to 40%. Although Hi-II performs very well at the tissue culture and transformation stages, T_0_ plants have poor seed setting capacity [158]. However, hybrids derived from the crosses between Hi-II and elite germplasm showed many “hybrid vigor (heterosis)” characteristics including more aggressive rooting, thicker stems, and taller stature than plants derived from Hi-II events. The hybrid T_0_ plants exhibited excellent tassel development in the glasshouse and the seed set was three to five times higher than Hi-II transformants [173]. Using standard binary vectors, an enhanced *Agrobacterium*-mediated transformation of Hi-II immature zygotic embryos was achieved recently by employing low-salt media in combined use with antioxidant l-cysteine alone or l-cysteine and dithiothreitol during the *Agrobacterium* infection stage [159].

### 6.2. Marker-Assisted Breeding for Transformability

Inbred line A188 has been shown to produce highly embryogenic callus in culture, leading to its frequent use in maize transformation investigations into the genetic control of embryogenic response in tissue culture. Early in 1992, Armstrong and Rout [160] proposed that there was a major gene (or genes) in the region marked by probe c595 on the long arm of chromosome 9 highly associated with several measures of in vitro culture response. Two independent mapping studies using A188 reported a total of seven QTL affecting tissue culture and transformability from A188. Willman et al. [174] suggested that at least one gene (or block of genes) controls the expression of the frequency of somatic embryogenesis. To further dissect the genetic basis of embryogenic response in maize, a mapping population of 101 RILs was developed from crossing A188 with a popular (nonembryogenic) maize inbred line, B73. The A188 × B73 (BC_3_S_5_) lines are estimated to contain approximately 3% of the A188 genome and 97% B73 [175]. Six lines were identified that produced a higher than expected number of somatic embryos when they were cultured for two weeks on a regeneration medium containing auxin, cytokinin and abscisic acid. At least one new locus was found that controls the production of somatic embryogenesis in A188. Lowe et al. [176] reported marker-assisted breeding (MAB) for transformability in maize. Maize lines with improved culturability and transformability were produced using MAB to introgress specific regions from the highly transformable hybrid, Hi-II, into the elite line, FBLL that responds very poorly in culture. FBLL is a female inbred parental stiff-stalk line that has been used in some historically best selling hybrids produced by a seed company DeKalb (now Monsanto). Five unlinked regions important for culturability and transformability were identified by segregation distortion analysis and introgressed into FBLL to produce the highly transformable FBLL-MAB lines.

### 6.3. Genetically Modified Maize

The commercial sector has made substantial progress with pest resistant maize through transformation with genes encoding for insecticidal crystal (Cry) proteins from *Bacillus thuringiensis* (Bt), which have been particularly successful in providing protection against several corn borers. Transgenic maize plants with the gene encoding snowdrop lectin gene under the control of a phloem-specific promoter were not only resistant to homopterns, but also showed toxicity to Asia corn borer, a type of Lepidoptera [108]. Transgenic plants with the *p1* transcription factor resulted in enhanced silk maysin production, thus achieving corn earwarm resistance as elevated concentration of silk maysin causes earworm abiosis [177]. 

There has been increasing interest in addressing more complex traits such as grain quality and abiotic stress tolerances. Naqvi et al. [178] created elite inbred South African transgenic corn plants in which the levels of three vitamins were increased specifically in the endosperm through simultaneous modification of three separate metabolic pathways. The kernels of the transgenic white corn (Cv. M37W) were found to contain 169-fold the normal amount of *β*-carotene, 6-fold the normal amount of ascorbate, and double the normal amount of folate. More examples for grain quality are discussed in the section on nutritional genomics**.** For chilling and cold tolerance, Ohta et al. [179] managed to shift the break point 3°C lower than that of the wild type by the introduction of an antisense gene for maize cold tolerant pyruvate orthophosphate dikinase (PPDK) into maize. Low level but constitutive expression of an active tobacco mitogen-activated protein kinase kinase kinase (Nicotiana *NPK1*) has been found to enhance freezing tolerance in transgenic maize plants that are normally frost sensitive [180]. The gene (NPk1) has been shown to have a significant effect on photosynthetic rates under drought stress when implemented with a modified constitutive promoter 35SC4PPDK [181]. As a consequence transgenic plants produced kernels with weights similar to those generated under well-watered conditions, while kernel weights of drought-stressed nontransgenic control plants were significantly reduced when compared with their nonstressed counterparts [182]. An elite maize inbred line, DH4866, has been transformed with the *beta A* gene encoding choline dehydrogenase, a key enzyme in the biosynthesis of glycine betaine from choline. The transgenic plants were more tolerant to drought stress than wild-type plants at both germination and young seedling stages. Most importantly, the yield was significantly higher than the wild type [183]. The same group has also produced and analyzed transgenic maize with improved salt tolerance through the introduction of *AtNHX 1* gene into maize genome, and some lines produced were able to germinate and grow in the presence of 0.8% and 1.0% sodium chloride [184]. Very recently, Castiglioni et al. [185] demonstrated that transgenic maize lines with bacterial RNA chaperones resulted in not only abiotic stress tolerance but also improved grain yield under water-limited conditions. The result supported the hypothesis that the endogenous function of cold shock proteins (CSPs) in plants relies on RNA binding/chaperone activity through the cold chock domain (CSD) and these proteins, similarly to bacteria, regulates stress responses through a posttranscriptional mechanism. A plant nuclear factor YB subunit (NF-YB) protein in *Arabidopsis * and an orthologous NF-YB protein from maize were identified coordinating plant responses to drought tolerance. The orthologous maize transcript factor, ZmNF-YB2, was shown to have an equivalent activity. Under water-limited conditions, transgenic maize plants with increased ZmNF-YB2 expression showed tolerance to drought based on the responses of a number of stress related parameters, including chlorophyll content, stomatal conductance, leaf temperature, reduced wilting, and maintenance of photosynthesis. These stress adaptations contributed to a grain yield advantage under water-limited environments. Under relatively severe conditions, the best performing transgenic maize line produced about 50% increase in yield relative to controls in the same experiment. The application of this technology has the potential to significantly impact maize production systems that experience drought [186].

### 6.4. Commercialization of Transgenic Maize and Its Impacts

Transgenic maize has been cultivated commercially in the United States since 1996. By 2000, about 25% of US maize had transgenic resistance to certain insects and/or herbicides, and this proportion increased to about 40% by 2003 [187] and 52% by 2005 [188]. Other countries that had approved releases of genetically modified (GM) maize by 1996 included Argentina and Canada. In Europe, GM maize was approved for use by the governments of Spain and France in 1998. Under European law, any seed which is approved in one EU country is automatically approved in all the others. But the process of extending approval for MON810 (resistance to European corn borer (*Ostrinia nubilalis*)) beyond France and Spain was suspended for five years by the EU moratorium on new GM products. The moratorium was lifted in May 2004, and the European Commission approved MON810 to be grown in any EU nation. But Greece still refuses to lift the ban on GM maize, while others such as Germany are providing approvals only on a case-by-case basis and primarily for nonfood uses.

Transgenic maizes producing insecticidal toxins from *Bacillus thuringiensis* (Bt) are widely used to control pests, but their benefits will be lost if pests evolve resistance [189]. The mandated high-dose/refuge strategy (www.epa.gov/pesticides/biopesticides/pips/bt_brad.htm) for delaying the development of resistance in the pest requires the planting of refuges of toxin-free crops near Bt crops to promote survival of susceptible pests who may have competitive advantage over resistant pests. To the contrary, resistance to a Bt crop has yet to be documented, suggesting that resistance management strategies have been effective thus far. However, current strategies to delay resistance remain far from ideal [190]. Unfortunately, it is highly difficult for governments in developing countries to enforce the refuge strategy. Moreover, it has been demonstrated that pollen-mediated gene flow (up to 31 m) from Bt maize caused low to moderate Bt toxin levels in kernels of nonBt maize refuge plants, which could seriously undermine the high-dose/refuge strategy and facilitate the accelerated development of pest populations resistance to Bt crops [189]. Thus, it now seems that farmers must implement measures to reduce gene flow between Bt crops and refuge plants. This is likely to be even more difficult than achieving widespread use of the refuge approach. Clearly, there is an urgent need to develop alternative ecologically and evolutionarily appropriate strategies that are easy to deploy in developing countries. 

Commercialization of transgenic maize for abiotic stresses such as drought tolerance has been very limited. There are several reasons for this. First, it is very difficult to phenotype the transgenics under either natural or controlled environments. Second, most of the comparisons between wild and transformed genotypes have been performed in conditions that are not very well reflective of developing country cropping environments. This has resulted in the identification and validation of transgene effects on drought tolerance under experimental conditions that are not mirrored under field conditions. This situation is changing now by an ongoing Water Efficient Maize for Africa project (http://www.aatf-africa.org/aatf_projects.php?sublevelone=30&subcat=5), which is a public-private partnership lead by African Agriculture Technology Foundation. In this project, CIMMYT will provide high-yielding maize varieties that are adapted to African conditions and expertise in conventional breeding and testing for drought tolerance. Monsanto will provide proprietary germplasm, advanced breeding tools and expertise, and drought-tolerance transgenes developed in collaboration with BASF.

Quist and Chapela [191, 192] reported the presence of transgenic DNA constructs in native maize landraces that were sampled from northern Oaxaca, Mexico. This raised questions about whether the introduction of large-scale commercial transgenic maize cultivar production would have a deleterious effect on the diversity of maize landraces and traditional agricultural systems of small-scale farmers. An important concern in assessing the risk of growing a genetically modified crop in its center of domestication is gene flow between the transgenic crop and its wild relatives. A second systematic survey of transgenes in currently grown landraces in the state of Oaxaca was carried out using highly sensitive PCR-based markers, appropriate positive and negative controls, and duplicate samples for DNA extraction. No transgenic sequences were observed so the report concluded that transgenic maize seeds were extremely rare (or entirely absent) in the sampled fields [187]. Cleveland et al. [193] analyzed the apparently conflicting conclusions from these two reports and sided with the original report. This was because the samples size used in the second study was not representative, and their statistical analysis was inconclusive due to using census population size (*n*) instead of effective population size (*N*_*e*_). Ortiz-García et al. [194] contested this finding and reiterated that there was a clear need for additional surveys with rigorous sampling methods to provide accurate estimates of transgene frequencies over broad geographic areas in Mexico. Large-scale chip-based whole genome fingerprinting, combined with pooled DNA analysis, would provide evidence for solving the conflicts.

## 7. Nutritional Genomics

In maize kernels, both macro- and micronutrients are present, including carbohydrates (starch), lipids and proteins (macronutrients), carotenoids, tocopherols, minerals, phytic acid, anthocyanins and other phenolic compounds (micronutrients). Malnutrition has long been recognized as a major public health problem in developing countries, including those where maize is used as staple food. Together with a balanced amino acid profile, attention has also been focused on three micronutrients where nutrient enhancement in maize could contribute to alleviate the problem: iron, vitamin A, and zinc [195]. In addition, the nutritional benefits of phytochemicals such as anthocyanins, xanthophylls, and tocopherols are also well recognized.

The term “nutritional genomics” is used to describe research and product development at the interface of plant biochemistry, genomics, and human nutrition [196]. It also involves the use of metabolic engineering, genetic engineering, and specific technologies. Despite many studies in these areas, there is still a lack of information for some traits, particularly in terms of isolation of regulatory elements and structural genes, duplicate function loci, feedback inhibition, branched pathways, or other phenomena affecting trait expression. Nevertheless, in the following subsections we present some examples of progress in understanding and manipulating phytic acid, iron, zinc, quality protein, and carotenoids.

### 7.1. Phytic Acid, Iron, and Zinc

Phytic acid, known as inositol hexakisphosphate (IP6) or phytate when in salt form, is a virtually ubiquitous component of plant seeds, supplying both phosphate and cations during germination. Phosporous bound in phytate is nutritionally unavailable to monogastric animals and thus contributes to water pollution because it is excreted in the waste [197]. In its native state, phytate forms complexes with proteins as well as mono- and divalent cations, thus decreasing the bioavailability of micronutrients like iron and zinc and exacerbating human mineral deficiencies. Although there are also positive effects against carcinogenesis that have been shown with in vitro cell cultures due to its antioxidant properties [198], phytate is considered an antinutritional compound [199]. Thus, the main advantage of *low phytic acid* (*lpa*) mutants is that the bioavailability of various minerals may be improved. However, due to its dual effect on human health, any strategy of reducing kernel phytic acid must consider the needs of the target population. 

Several mutants with low levels of phytate have been isolated, and the loci mapped in maize, including *lpa 1-1*, *lpa 2-1, * and * lpa 241* [200, 201]. Several studies have been conducted to understand phytic acid biosynthesis, identifying genes involved in the pathway. Using *Mutator* insertion knockout technology, Shi et al. [202] identified a maize inositol phosphate kinase gene involved in phytic acid biosynthesis in developing seeds. The ZmIpk loss-of-function mutant for this gene is allelic to the low-phytic acid mutant *lpa2*. Cloning and sequencing of the ZmIpk gene from *lpa2-2* showed that the *lpa2-2* allele has a nucleotide mutation that causes immature termination of the ZmIpk open reading frame. In the *lpa2-1* mutant, the genomic sequence was found rearranged in the ZmIpk locus, and no mRNA expression was detected [202]. Despite efforts to elucidate and manipulate phytic acid biosynthesis, low-phytic acid mutants have limited value to breeders because of adverse effects on agronomic traits such as low germination rates, reduced seed weight (*lpa1-1*), stunted vegetative growth and impaired seed development (*lpa241*). However, Shi et al. [203] have recently identified the gene disrupted in maize *lpa1* mutants as a multidrug resistance-associated protein (MRP) ATP-binding cassette (ABC) transporter. Silencing expression of this transporter using the embryo-specific Glb promoter produced low-phytic acid, high phosphate transgenic maize seeds that germinate normally and do not show any significant reduction in seed dry weight. 

To increase the amount of bioavailable iron in maize, Drakakaki et al. [204] have generated transgenic maize plants expressing aspergillus phytase and iron-binding protein ferritin. This strategy has proven effective for increasing iron availability and enhancing its absorption. However, much work is still to be done to transfer this technology to tropical and subtropical maize genotypes normally grown in the areas of greatest need for enhanced iron content maize. 

Due to the complex and still poorly understood action of iron and zinc in plant metabolism, not much work has been carried out on their nutritional genomics in maize. A bioinformatics approach, however, has been reported by Chauhan [205] toward the identification of candidate genes for zinc and iron transporters in maize using the sequence data available for maize and the iron and zinc metabolism information derived from other plant species such as *Arabidopsis*.

Conventional biofortification breeding efforts are attempting to enhance iron and zinc content by using maize genetic resources. At CIMMYT, we have analyzed more than 1000 improved maize genotypes and 400 landraces from different environments. However, we found little variation in grain for iron levels (average 20 ± 5 ppm) [206, 207] and only moderate variation for zinc concentration in grain (15–35 ppm).

### 7.2. Protein

The major maize seed storage proteins, zeins, are deficient in two essential amino acids, lysine and tryptophan, and therefore contribute to the poor nutritional quality of maize. Kernels with reduced levels of zein proteins (through mutation or genetic engineering) have been shown to have increased levels of lysine and tryptophan [208]. The presence of a naturally-occurring mutant gene, the *opaque-2* (*o2*), results in increased concentrations of lysine and tryptophan in maize grain, which has been named quality protein maize (QPM). This equates to a biological nutritional value for QPM protein that is 90% equivalent to that of protein in milk, whereas that of regular maize grain in only about 40% [209]. 

Gene expression studies, QTL analysis, and progress in proteomics, transcriptomics and conventional breeding have helped to elucidate the QPM trait [208, 210]. Breeding of QPM cultivars requires manipulation of three genetic systems: (1) the *opaque-2* (*o2*) gene must be in its homozygous recessive form, thereby reducing the rate of transcription of genes encoding zein proteins, which contain very small quantities of lysine and tryptophan; (2) modifier genes of the *o2* gene must be selected to modify the undesirable soft and chalky (opaque) kernel features that are typical of *opaque-2* maize; (3) additional (non-*o2*) genes affecting lysine and tryptophan concentration in grain must be selected to ensure that concentrations of these amino acids are within the high range of variation observed for maize [211, 212].

By genetic engineering, a dominant opaque phenotype has been obtained by reduction of the zein proteins in the grain showing increased lysine and tryptophan contents [213, 214]. Most recently, Houmard et al. [215] have reported the increase of lysine in maize grains by endosperm-specific suppression of lysine catabolism using RNA interference (RNAi). A different approach to increase protein quality in maize using genetic engineering has been the introduction of the gene encoding amarantin. Amarantin is a protein from the *Amaranth* plant, which is known to be well balanced in its amino acid content. An increase of 8% to 44% in essential amino acids was observed for maize transformed with the amarantin encoding gene [216]. However, the efficacy of this maize for target population is still to be demonstrated.

### 7.3. Carotenoids

Given the general nutritional interest in carotenoids for intermediate and end products of maize, the carotenoid metabolic pathway has been intensively researched. All yellow maize contains carotenoids, although the fraction of carotenoids with provitamin A activity (*β*-cryptoxanthin, *α*- and *β*-carotene, which can be converted to vitamin A) is typically small (15% to 18% of the total carotenoid fraction) compared to zeaxanthin and lutein (around 45% and 35%, respectively) [217, 218]. Due to the general vitamin A deficiency in developing countries, efforts have been concentrated on increasing the amount of provitamin A carotenoids in staple crops including maize [219]. There is considerable scope for breeding maize with enhanced provitamin A concentrations by shifting carotenoid biosynthesis to favour provitamin A versus other types of carotenoids, and hence enhancing nutritional value [218, 220, 221].

Analyses of genotypes with yellow to dark orange kernels have identified large variation in the number of provitamin A molecules [220, 222] and their carotenoid profiles. At CIMMYT, we have analyzed carotenoid profiles for more than 1000 tropical genotypes and identified promising materials with provitamin A concentrations (ca. 8 *μ*g/g) and/or carotenoid profiles that could be used in breeding progams to increase total provitamin A content in maize grain. To date we have observed no consistent trend in the origin of maize genotypes with the highest provitamin A concentrations; the best materials include pale-yellow temperate, dark-yellow highland tropical, and intense-orange tropical lines. 

There are many carotenoid phenotypic mutants in maize that have been associated with cloned genes. *Psy1* (phytoene synthase) was cloned by transposon tagging and mapped to chromosome 6 [223], and in the presence of the resulting Yellow 1 (Y1) gene product, carotenoids are produced in the endosperm tissue, yielding the yellow endosperm phenotype. The other allelic form and *psy2* are not expressed in endosperm [72, 223, 224]. In addition, QTL analyses have shown that candidate genes *Psy*1 and *Zetacarotene desaturase* (*Zds*) are associated with variation of individual and total carotenoid contents [225]. 

Using an RIL population derived from a cross between By804 and B73, 31 QTL including 23 for individual and eight for total carotenoids were detected [226]. Much of the phenotypic variation in carotenoids could be explained by two loci, and the QTL for carotenoids elucidated the interrelationship among these compounds at the molecular level. A gene targeted marker in the candidate gene *psy1* tightly linked to a major QTL was identified explaining 6.6%–27.2% of phenotypic variation for levels of carotenoids. Fraser and Bramley [227] confirmed that *LycE* controlled the ratio of carotenoids in zeaxanthin (with provitamin A intermediates) and lutein branches, being a key enzyme in the provitamin A content of maize. Through association analysis, linkage mapping, expression analysis, and mutagensis, Harjes et al. [29] showed that variation at the *lycopene epsilon cyclase* (*lcyE*) locus alters flux down *α*-carotene versus *β*-carotene branches of the carotenoid pathway. Four natural *lcyE* polymorphisms explained 58% of the variation in these branches and a threefold difference in provitamin A compounds. Another gene in the pathway, *carotene hydroxylase enzyme* (*CrtR-B1*), has also been cloned and analyzed (Jianbing Yan, unpublished). A recent report in genetic transformation, as discussed in Section 6, has used particle bombardment to generate multivitamin maize with significantly increased contents for *β*-carotene, ascorbate, and folate [178]. This is a very important proof of concept for genetic manipulation of distinct metabolic pathways. However, appropriate and fast strategies have to be developed now to obtain the useable products and the desired impact in the target countries.

## 8. Genomics-Assisted Breeding

While almost all maize plants grown in the fields of developed countries are hybrids, various types of maize cultivars are grown in developing countries, including hybrids, synthetic cultivars, open pollinated varieties (OPVs), and landraces. In addition to the target type of maize cultivar, breeding objectives are dominated by the target environment. High yield potential and good grain quality are primary selection criteria for maize breeding programs across both tropical and temperate areas. However, tropical maize breeders are required to address some very specific abiotic and biotic stress tolerance objectives that are rarely important for temperate maize breeding programs.

The multinational seed companies are now routinely using applied genomic tools to (i) dissect the genetic structure of relevant maize germplasm to understand gene pools and germplasm (heterotic) groups, (ii) provide insights into allelic content of genetic resources for potential use in breeding, (iii) screen early generation breeding populations to select segregants with desired combinations of marker alleles associated with beneficial traits (in order to reduce the scale of costly phenotypic evaluations), (iv) pyramid favorable genes/alleles from different germplasm sources through marker-assisted recurrent selection in order to improve genetic gains, and (v) establish genetic identity (fingerprinting) of their products [61, 228–235]. MAS has been successfully applied by private sector maize cultivar development aimed at recovery of an ideal genotype defined as a mosaic of favorable chromosomal segments. Parental genotypes are selected to provide specific components of this mosaic including favorable alleles for multiple complex traits such as yield, biotic and abiotic stress resistance, and quality attributes [234, 236–238]. Using these approaches, the commercial breeding programs report a doubling in the rate of genetic gain over phenotypic selection (PS) in maize [233, 237]. 

A large proportion of the successful reports of MAS in maize have involved MABC or advanced backcross QTL (AB-QTL) for introgression of favorable alleles (foreground selection) and for accelerating the recovery of the recipient genotype in the remainder of genome (background selection, see Table 1). In theory, the relatively easy target traits for MAS include grain quality genes and major disease resistance genes. However, there are also some reports regarding complex traits. A good example was the improvement of the B73 × Mo17 hybrid through marker-assisted enhancement with elite alleles from Tx303 and Oh43 [239]. On the basis of four years of testing, yields of the best “enhanced” B73 × enhanced Mo17 hybrids exceeded the original hybrid and high yielding commercial hybrids by 8% to 10%. In another case, the drought susceptible line CML247 was improved with five favorable alleles from the drought tolerant line Ac7634 through four generations of MABC. As a result, the best five MABC-derived hybrids yielded, on average, at least 50% more than the control hybrids under water stress conditions [240, 241]. 

Despite all the recent technological breakthroughs, there are limited reports on the overall contribution of genomics-assisted breeding of quantitative traits in released cultivars, particularly in public sector breeding programs [61, 242]. Progress in rice has been better documented in literature [243]. For example, a gene underlying a large-effect QTL contributing to submergence tolerance in rice has been cloned and introgressed into a wide range of cultivars [244]. Although there is very limited specific information on the successes of molecular breeding, the first commercial products of molecular breeding (rather than limited MAS) are already being released to the market by several major multinational breeding companies. The first hybrid maize cultivars developed through molecular breeding by Monsanto were released for commercial production in the USA for the 2006 cropping season, and it is estimated that, by 2010, over 12% of the commercial crop in the USA will be derived from products of molecular breeding [245].

### 8.1. Yield and Heterosis

Dominance, overdominance, and epistasis have all been proposed to have a role in the genetic basis of superior hybrid performance. The dominance model attributes increased vigor to the interaction of favorable dominant alleles from both parents when combined in the hybrid [246], whereas the overdominance model postulates the existence of loci at which the heterozygous state is superior to either homozygote [52]. Evidence for the role of epistasis (interaction of the favorable alleles at different loci contributed by the two parents) in hybrid vigor has also been reported [247–249]. The genetic basis of heterosis, heterotic groups, hybrid prediction and hybrid performance, relationships between heterozygosity and genetic distance with hybrid performance and heterosis, and use of MAS in hybrid breeding has been reviewed elsewhere [229].

Harnessing heterosis has been a corner stone of maize breeding for nearly a century and has been more extensively used than for any other crop. Therefore, development of a reliable method for predicting hybrid maize performance without generating and testing hundreds or thousands of test-cross combinations has been the goal of numerous studies, using both marker data and a combination of marker and phenotypic data [239]. Most of these studies concentrated on probing the general correlation between genetic distances revealed by molecular markers and hybrid performance. However, without identifying the association of markers with specific genes for hybrid vigor and performance, the correlation revealed by random markers has only been able to provide very limited predictive power. 

The definition of heterotic groups and heterotic patterns in temperate hybrid maize breeding has contributed to large increases in yield [250]. Reciprocal recurrent selection breeding programs have proven effective in the improvement of heterotic groups for a systematic exploitation of heterosis, as they maximize selection gains within a heterotic group while at the same time maximizing differences between heterotic groups. Clear characterization of genetic diversity among maize inbred lines derived from different origins can increase the efficiency of predicting good hybrid combinations for developing new inbred lines. In temperate maize such as the US Corn Belt germplasm, a clear heterotic pattern (Reid Stiff Stalk versus Lancaster) was established early on and inbred lines such as B73 and Mo17 from the two heterotic groups were chosen as testers for the selection of new maize inbred lines. Today two heterotic groups (Stiff-Stalk (SS) versus Non-Stiff-Stalk (NSS)) are clearly distinguished. For tropical germplasm, there are two apparent heterotic groups: SS-Tuxpeno versus NSS-Non-Tuxpeno. 

Following successful deployment of hundreds of OPVs in the 1970s and early 1980s, the CIMMYT maize program began the development of hybrid maize to meet the needs of an increased number of developing country farmers who were eager to switch to this type of variety. In the 1990s, 10 pairs of heterotically distinct genotypes were identified or generating in subtropical, midaltitude, and highland populations to provide heterotic pools for breeders targeting each of these environments. More recently, testers from each population have been used to identify the hybrid performance of inbreds from the partner populations and help assign new inbred lines to appropriate heterotic groups.

Molecular markers are useful for helping to define heterotic groups and to examine the relationships among inbred lines at the DNA level. Various types of molecular marker have been used to investigate relationships among inbred maize lines from different heterotic groups, including tropical maize [13]. Markers are also useful to assign new lines to new or currently existing heterotic groups.

### 8.2. Abiotic Stresses

New molecular marker technologies and comprehensive gene expression profiling methods provide opportunities to direct the breeding of improved genotypes that provide stable grain yield under varied suboptimum environmental conditions [251]. In this context physiological genomics may improve our ability to manipulate the genome of crop plants in order to improve their adaptation to stresses [252, 253]. 

QTL analysis has improved our knowledge of the genetic basis of a number of morphophysiological traits involved in the response to different abiotic stresses in maize. A number of candidate genes mapped near to QTL regulating important morphophysiological traits and grain yield have already been identified in maize. An updated compilation of mapped QTL and major genes associated with abiotic stress tolerance in maize and other plants is available at http://www.plantstress.com/biotech/index.asp?Flag=1. Among the most important traits are yield, flowering and phenological parameters, plant height, ear number, photosynthesis, chlorophyll fluorescence and leaf ABA concentration, and root traits such as adventitious root formation for waterlogging and chilling tolerance as well as root hair traits for phosphorus deficiency and aluminum tolerance. Khavkin and Coe [264] hypothesized that many apparent QTL of major effect in maize are in fact clusters of genes (e.g., homeotic genes and other genes encoding for transcription factors) regulating development and that many plant reactions to abiotic stresses rely on such gene clusters.

Drought tolerance has been the most difficult abiotic stress for breeders to make rapid, substantial, and consistent advances. The genetic basis of the molecular, cellular, and developmental responses to drought involves many gene functions regulated by water availability [242]. In that context, genomics-based approaches could help access to agronomically desirable alleles present at QTL which eventually may help to improve the drought tolerance and yield of crops under drought. The identification and cloning of genes at target QTL may further broaden our understanding of the genetic, physiological, and functional bases of drought tolerance [265].

A major effect of water stress in maize is a delay in silking, resulting in an increase in the anthesis-silking interval (ASI), which leads to substantial yield reduction or even complete crop failure. Thus, developing maize lines with a short ASI has been an important goal for drought tolerance breeders. QTL influencing flowering time and ASI have been identified [266–268] and the availability of molecular markers linked to five QTL for ASI enabled lines with a reduced ASI to be selected [55, 240]; however, the yield advantage was only evident under very severe stress conditions. In addition, QTL for silking date, grain yield and yield stability under field conditions have been reported [269]. Since vegetative plant growth is strongly affected by drought stress, emphasis has also been placed on identification of QTL for the response of leaf elongation rate to soil moisture, temperature and evaporative demand [270]. Several factors appear to have confounded the detection of QTL in maize that could be useful in marker-assisted development of drought-tolerant cultivars. Among them has been a tendency to examine crosses between lines that were not agronomically elite and/or did not exhibit extreme differences in yield under stress. Furthermore, many studies have used relatively small mapping populations and have achieved low precision in phenotyping, factors that are well known to result in low power of QTL detection and severe bias in the estimation of QTL effects [271]. Other studies have relied on an inadequate phenotyping in terms of how, when and what traits are measured, thereby limiting their impact [272]. Thus, the use of MAS for improving complex traits remains a challenge for crop breeders [273], at least in the public sector [61]. While the genetic dissection of crop performance in drought-prone environments has greatly benefited from the use of DNA markers [241, 242, 274, 275], the outputs have not been routinely implementation in practical breeding programs. QTL are often germplasm-specific and the costs for applying MAS for many QTL of small effect may be greater than those of conventional cross-breeding. The challenge for molecular breeders is to identify QTL of major effect that are independent of genetic background and to devise more effective breeding approaches for the application of the resultant markers, such as pedigree selection. This approach has shown promise in rice [276, 277] as well as in durum wheat [275]. In both cases, results suggest that breeding for traits affecting yield potential can be translated into better performance under drought stress [265]. 

MAS for drought-related traits based on genetic mapping information should preferably target “major” QTL with a sizeable effect, consistent across germplasm and with a limited interaction with the profile of water availability. In maize, however, QTL studies in the past have not identified any QTL with sufficiently large effects to be effectively used in MAS programs. Marker-assisted recurrent selection provides an alternative approach to improve drought tolerance through genome-wide selection or selection based on an index constructed from a set of markers associated with different traits or trait components. This approach has been proven to be successful in private sector breeding programs [234, 235] and is being extensively implemented in the Drought Tolerance Maize for Africa project using chip-based SNP markers (http://dtma.cimmyt.org).

### 8.3. Quality

The molecular breeding of QPM has been reviewed elsewhere [278]. There are three markers available for MAS in QPM breeding (phi057, phi112, and umc 1066). One of the markers (phi112) provides only dominant information, and thus can only be used to identify genotypes that do not contain a recessive *o2* allele. The other two markers exhibit codominant polymorphism between normal and QPM inbreds [262]. Even with MAS, due to the polygenic nature of QPM and the current status of available markers, protein content and quality must still be monitored using biochemical methods. The most difficult aspect of QPM molecular breeding is the selection for minor loci controlling modification of lysine and tryptophan levels in *o2o2* backgrounds, of which over eight loci have been mapped in various studies [212]. At least two and likely more loci affect the modification of the endosperm hardness in *o2o2* backgrounds [279]. Several major *o2 * modifier QTL have been mapped to chromosomes 1, 7, and 9. A microarray hybridization performed with RNA obtained from true breeding *o2* progeny with vitreous and opaque kernel phenotypes identified a small group of differentially expressed genes, some of which mapped at or near the *o2* modifier QTL. Several of the genes were associated with ethylene and ABA signaling which suggests a potential linkage of *o2* endosperm modification with programmed cell death [280].

Using SSR-based MABC breeding, maize lines have been developed that have twice the amount of lysine and tryptophan than the native lines and up to 95% of the recurrent parent genome [262]. In African maize breeding, *o2* allele specific SSR markers were used to convert herbicide resistance maize lines into QPM which is the equivalent of modified *o2* phenotype. Using the three SSR markers, the result showed that 97% of the lines were *o2*. Conventional methods using a light table or light box and MAS obtained comparable results [263]. However, application of SSR markers and the FTA DNA extraction technology offered the breeder a fast, reliable and less labor intensive method of screening QPM maize during the early growing stages instead of having to wait to screen the kernels on the light table after harvesting. Unfortunately, o2 selection alone is not sufficient as breeders must also select for the tryptophan and lysine modifiers using biochemical analysis. Using a new high lysine mutant, *o16*, which contains similar levels of lysine to *o2* mutants [281], MAS for combining favorable alleles of both *o2* and *o16* would facilitate development of new high lysine maize cultivars once the genetic effect of *o16 * is confirmed under different genetic backgrounds [278].

Plant oils have been receiving increasing attention as an important renewable resource for biodiesel production and for dietary consumption by humans and livestock. A high-oil QTL (*qHO6*) affecting maize seed oil and oleic-acid content has been shown to encode an acyl-CoA : diacylglycerol acyltransferase (*DGAT1-2*), which catalyzes the final step of oil synthesis. A phenylalanine insertion in *DGAT1-2* at position 469 (F469) was responsible for the increased oil and oleic-acid content. Ectopic expression of the high-oil *DGAT1-2* allele increased oil and oleic-acid content by up to 41% and 107%, respectively [282]. This work provides insights into the molecular basis of natural variation of oil and oleic-acid contents in plants and highlights *DGAT* as a promising target for increasing oil and oleic-acid content in other crops.

MAS and phenotypic selection (PS) were applied to three F_2:3_ populations as base (starting) populations (*C*_0_) with either the *su1*, *se1*, or *sh2* endosperm mutations [283], which are related to sweetcorn quality. Of the 52 paired comparisons made between composite populations derived through MAS or PS, MAS resulted in significantly higher gains than PS in 38% of comparisons across the three *C*_1_ composite populations, while PS was significantly greater in only 4% of the comparisons. The average MAS and PS gain across all composite populations and selected traits, calculated as a percentage increase from the randomly selected controls, was 10.9% and 6.1%, respectively. Another example is the development of an MAS system for two genes in the carotenoid pathway that have been cloned (as discussed in the previous section). Inexpensive PCR-based markers for provitamin A are being developed and validated at CIMMYT from the cloned gene sequence (unpublished results, CIMMYT) using seed DNA-based genotyping method [284], which will enable developing-country breeders to more effectively produce maize cultivars with higher provitamin A levels. A molecular breeding platform based on MAS using SNP-chips has been developed and used for linkage mapping and LD-based analysis and molecular breeding for both qualitative and quantitative traits [86, 285].

## 9. Genomic Databases and Informatics Tools

In the past few years, several important databases have been built focused on data from maize genomics research (Table 2). One of the most important collection of genomic databases and informatics tools is MaizeGDB (http://www.maizegdb.org). Among the data sets included in MaizeGDB are sequences, including integration with various contig assemblies; publication references; detailed genetic, physical, and cytogenetic maps; QTL mapping results; mutants; genes; primers; and a wealth of other data types. MaizeGDB includes integrated tools for map comparisons, sequence similarity searches, and comparisons with and links to other databases, such as Gramene and NCBI. MaizeGDB provides web-based curation tools that enable researchers to edit and annotate their own data and to enter new data into MaizeGDB directly. MaizeGDB also provides informatics support for maize community initiatives such as the annual Maize Genetics Conference and community-wide workshops, and maintains data for maize community research projects. To have easy access to systematized information about all known QTL for traits of interest, coupled to other information about germplasm, nearby loci and sequence information, MaizeGDB began curating QTL information from literature in the mid 90's. To permit this work to continue at MaizeGDB, a new Web accessible curation interface has been designed and implemented. The new design accommodates a legacy trait hierarchy developed at MaizeGDB and recently harmonized with the rice Trait Ontology at Gramene, and trait descriptors used by GRIN (the Germplasm Resources Information Network).

The Maize Assembled Genomic Island site (MAGI, http://magi.plantgenomics.iastate.edu) is a resource for maize genome assembly, annotation and mapping, which assembled a large number of maize genomic sequences primarily composed of gene-enriched GSSs (genomic survey sequences), random Whole Genome Shotgun (WGS) sequences, and BAC shotgun reads [286]. GBrowse, a component of GMOD (a Generic Model Organism Database Toolkit), is used to display annotated assemblies. The MAGI website can serve as a community resource for map-based cloning projects as well as for analyses of genome structure and comparative genomics. The FPC resources at the Arizona Genomics Institute provide information on the status of the agarose FPC map. WebFPC and its associated tools provide online access to the contigs. Much effort on maize genome sequence annotation has been made at http://www.maizesequence.org, linking maize sequence to physical and genetic maps, and providing computational annotation of predicted genes—and this portal will see widespread use as the sequence is completed and groups start to focus on functional analysis.

To enable biologists to simultaneously query phenotype data by image example, sequence, ontology, genetic and physical map information, and text annotations, a web-based visual phenotypic information management system, VPhenoDBS (medbio.cecs.missouri.edu/VPhenoDBS), is being developed [287]. The database framework consists of five modules: a system to extract and quantify low level features from phenotypic images, a high-dimensional database indexing system to manage and cluster images for real-time retrievals, a linking hub to correlate visual features already attributed to a given locus with relevant genetic and physical maps, a text mining and ontology utilization system for parsing annotations, and a results visualization system. This system may be integrated with a fully automatic high-throughput screening system, the *Scanalyzer 3D,* as presented for complete plants like maize, rice, *Arabidopsis*, poplar tree, barley or wheat in the greenhouse, combining information from all 3 dimensions (http://www.lemnatec.de/scanalyzer_gh.htm). This screening system is able to 3-dimensionally screen up to 4000 plants per day efficiently and precisely. With the *Scanalyzer 3D* a wide range of visual evaluation parameters of plants can be sampled for a complete, reproducible and nondestructive analysis free of subjective influences.

There are many software and decision support tools developed in plant genomics including those for germplasm evaluation, breeding population management, genetic map construction, marker-trait association analysis, MAS, GEI analysis, breeding design and simulation, and information management [288]. Table 3 lists some tools, which include QTL meta-analysis, QTL and gene comparative analysis, population structure and kinship evaluation, and association mapping that are particularly valuable in maize genomics research and molecular breeding.

Two software packages that are more specific to maize will be discussed here. The first is TEnest, which was developed to facilitate identifying repetitive sequences and reconstructing separated sections to provide full-length repeats and, for long-terminal repeat (LTR) retrotransposons, calculating age since insertion based on LTR divergence [289]. Considering that 85% of the maize genome consists of transposable elements (TEs), with more than 70% of TEs found nested within one another, an accurate nested TE identification tool for complete annotation of the maize genome is needed. TEnest contains an up-to-date database of maize canonical TEs and their associated LTRs, if applicable. The second software we would like to highlight is an integrated software for SNP discovery in maize [290]. The development of software tools to aid researchers in the SNP discovery process across several maize, teosinte, and *Tripsacum* lines has been the focus. An integrated set of tools consisting of a relational database and applications for data loading, editing and reporting has been developed (http://www.panzea.org). All stages of SNP discovery from tracking sequences, alignment generation, alignment editing, and reporting are covered. Central to this system is an intuitive, quality score-based alignment editing tool designed to simplify manual editing of the highly polymorphic and complex *Zea* alignments.

## 10. Outlook

Genome-wide scans for genetic mapping and whole genome sequence-assisted marker development and application have now become possible in maize. The process will be accelerated by high-throughput technologies for both phenotyping and genotyping, and will be facilitated by bioinformatics and decision support tools [61]. We anticipate that large-scale genomics-assisted marker development and gene discovery will be routinely applied in both private and public sectors for traits of economical importance, especially where individual genes or QTL of large effects can be identified that are not significantly influenced by environmental factors. Conversely, to make improvements in traits controlled by many genes, each having minor effects and all having large GEI, will require a much more complicated approach. In particular, well adjusted strategies and strengthened multidisciplinary collaboration will be needed, and in many cases the solutions will be trait-specific in development and population-specific in application.

Considering the progress in the various “omics” areas and the integration of different disciplinary applications facilitated by bioinformatics, as well as high-throughput genotyping approaches combined with automation, MAS will gradually evolve into more holistic “genomics-assisted” breeding strategies. Since genomics resources in maize are among the best of the major crop species, the role of genomics is set to become more and more important in maize breeding. However, conventional selection will remain a vital element of the process to finally confirm the best candidate genotypes for progression into the advanced stages of crop improvement and cultivar selection. Thus, it will not be a case of MAS replacing parts of traditional breeding programs, but rather an increasing reliance on genomics data alongside other technology interventions in an ever evolving and refining breeding system. Genomics-assisted breeding systems will be evaluated in terms of their ability to increase the scope of breeding goals, to provide new added-value traits, to decrease the cost of breeding programs, and to improve the pace of developing new cultivars, and finally to enhance impact of resultant products to command increasing areas of production. 

Transcriptomics is a field that may experience significant innovations in the near future with increasing impact on physiological studies of maize. Monitoring large-scale changes in transcript profiles may eventually allow for the identification of transcript networks accounting for GEIs in different genotypes [291]. However, there are several major limitations of microarray-based expressional studies, for example: (1) low-abundance mRNA may not be represented by most arrays nor detected upon hybridization; (2) the correlation between the level of mRNA and the products of their translation or their biological effect can be low. Genome-wide transcript profiling may encounter a similar history as genome mapping that started with great impacts for the traits controlled by major genes or QTL without significant GEI, but still faces challenges for manipulating complex traits.

Maize as a multipurpose crop of importance in all global crop production regions will continue to play a leading role in shaping the future of crop improvement and production systems. Advances in maize genomics, breeding and production will have significant impact on the lives of a large proportion of the world's population [292]. Balancing consumer demand for various usages of maize with different maize production practices will be critical for maintaining both sustainability of cropping systems, food security, feed and fodder supply and bioenergy demands. It is important to note that the edible portions of food crops are not the most desirable plant portion for creating biofuel in many plants including maize. The current processes of generating biofuel from maize must be improved either by more efficient use of the maize grain or better conversion of cellulose contained in the stalks into biofuels. The latter, so-called second generation biofuels, have not yet been fully developed for any crop but hold much promise. Of particular importance is that this approach would not directly compete with much-needed food supply. Although it would compete with sustainable conservation agriculture production systems and the increasing demand for fodder to support the rapidly expanding livestock industry in developing countries. There is clearly no quick and easy fix to this dynamic situation and much careful thought and action will be required to properly manage the situation [293].

As maize is a fundamentally important commodity in both developed and developing countries and used in many varied ways, North-South collaborations in maize genomics should be strengthened for scientists in both theoretical and applied genomics fields. We can safely assume that the rapid developments in maize genetics and genomics, although currently based mainly on temperate maize germplasm, will be transferable and increasingly valuable for improvement of tropical and subtropical maize which is the important crop for food security in developing countries. With the resolution of many practical, logistical and genetic bottlenecks in MAS [61], including development of seed DNA-based genotyping [284], and the ongoing development of powerful decision support tools, it can be expected that genomics-assisted approaches will increasingly become a routine component of breeding programs of private and public sectors worldwide.

## Figures and Tables

**Table 1:** Examples of MAS applications in maize improvement.

Traits	Molecular markers	Note	References
Grain yield, ear number, plant height	RFLP	Increase of hybrid yield by enhancing both parents with introgression of favorable alleles	[239, 254]

Grain yielding, percent stalk lodging	RFLP	Early generation test of inbreeding process	[255]

Grain yield, earliness	RFLP	Foreground selection for three loci of earliness and grain yield and background selection	[256]

Grain yield	SSR	Four yield QTL were selected as target introgressions in the developmentof BC3 families	[257]

Seeding emergency	RFLP	Seedling emergence of three elite commercial sweet corn inbreds has been improved by introgression of three alleles with marker-assisted backcross	[258]

Drought tolerance	RFLP, SSR	Five favorable alleles related to drought tolerance were successfully introgressed into the recurrent parent	[240]

Fertility restorer gene	SSR	Selection of *Rf3* gene	[259]

Southwestern corn borer	RFLP	A total of five loci traced during backcross	[260]

Stalk strength, second-generation European corn borer	RFLP	Comparison of effectiveness between phenotypic selection and MAS on stalk strength and second-generation European corn borer resistance	[261]

Quality protein maize (QPM)	SSR	Both foreground selection for the *o2 *gene and background selection for restoration of recurrent genome	[262]

Quality protein maize (QPM)	SSR	*o2* gene was introgressed into herbicide resistant elite maize inbred lines using three SSR markers	[263]

Drought tolerance	RFLP	Inbred line CML247 was improved for drought tolerance through four cycles of MABC	[241]

**Table 2:** Databases and resources related to maize.

Name and link	Description
*MaizeGDB * http://www.maizegdb.org/	A community database for biological information about the crop plant *Zea mays* ssp. mays. Genetic, genomic, sequence, gene product, functional characterization, literature reference, and person/organization contact information are among the data types accessible (see Section 9 for further details)

*MAGI * http://magi.plantgenomics.iastate.edu/	Maize assembled genomic island (MAGI) reports the results of a maize genome assembly project being conducted by the Aluru, Ashlock and Schnable research groups

*Gramene * http://www.gramene.org/	A resource for comparative grass genomics including: genomes, protein, maps, markers, traits, genetics diversity, biochemical pathway, literature, and so forth

*Maizesequence * http://www.maizesequence.org/index.html	Provides sequence and annotation of the *Zea mays * ssp. Mays genome resulting from the maize genome sequencing project

*mtmDB * http://mtm.cshl.edu/	A transposon library in maize for the purpose of targeted selection of gene knockouts

*Maize tilling * http://genome.purdue.edu/maizetilling/	A website for maize tilling project that collects and distributes maize tilling mutant and seeds

*NCBI Maize * http://www.ncbi.nlm.nih.gov/mapview/map_search.cgi?taxid=4577	Provides all of the accessions from *Zea mays* including: genomics, cDNA, EST, GSS, HTG sequences

*SAM * http://sam.truman.edu/	A database for maize shoot apical meristem expression

*Helitons * http://genomecluster.secs.oakland.edu/helitrons/	A catalog of discovered helitrons

*Grassius * http://www.grassius.org/	Provides a collection of databases for transcription factors, promoters and cis-regulatory elements across the grasses, including maize, sugarcane, sorghum, and rice

*Panzea * http://www.panzea.org/	A database for molecular and functional diversity of the maize genome

*MaizePLEX * http://www.plexdb.org/plex.php?database=Corn	A miame-compliant and plant ontology enhanced expression database for maize microarray data

*TIGR Maize * http://maize.tigr.org/	Provides links to the NSF-funded consortium for maize genomics project and includes sequence, assembly, and annotation data, and links to the maize gene index

*MaizecDNA * http://www.maizecdna.org/	Released 30000 FLcDNA from diverse B73 tissues samples

*ZmGDB * http://www.plantgdb.org/ZmGDB/	Provides a convenient sequence-centered genome view for *Zea mays*, with a narrow focus on gene structure annotation

*PML * http://chloroplast.uoregon.edu/	A genetic resource in maize, that is, tailored for studies of chloroplast biogenesis

*VPhenoDBS * http://medbio.cecs.missouri.edu/VPhenoDBS/	Web-based visual phenotypic information management system to allow simultaneously query phenotype data by image example, sequence, ontology, genetic and physical map information, and text annotation

**Table 3:** Bioinformatics tools for maize genomic and functional research.

Name and link	Description
*MetaQTL * http://bioinformatics.org/mqtl/	A java package designed to perform the integration of data from the field for gene mapping experiments

*BioMercator * http://cms.moulon.inra.fr/index.php?option=com_content&task=view&id=13&Itemid=43	Genetic maps and QTL integration

*CMTV * http://www.ncgr.org/cmtv/	An integrated bioinformatics tool to construct consensus maps and compare QTL and functional genomics data across genomes and experiments

*QTL-Finder * http://gqtl.maizecenter.cn	A bioinformatics tool for QTL integration, comparison and candidate gene discovery across genomes and experiments

*TWINSCAN * http://mblab.wustl.edu/query.html	Improved gene prediction performance for maize and rice

*GDPC * http://www.maizegenetics.net/gdpc/index.html	The genomic diversity and phenotype connection (GDPC) simplifies access to genomic diversity and phenotype data, thereby encouraging reuse of this data. GDPC accomplishes this by retrieving data from one or more data sources and by allowing researchers to analyze integrated data in a standard format. GDPC provides access to genomic diversity data such as SNPs, SSRs, and sequences, and phenotypic data that may be collected in field, genetic, or physiological experiments

*TASSEL * http://www.maizegenetics.net/index.php?page=bioinformatics/tassel/index.html	A software package to evaluate trait associations, evolutionary patterns, and linkage disequilibrium

*PowerMarker * http://statgen.ncsu.edu/powermarker/	A comprehensive set of statistical methods for genetic marker data analysis, designed especially for SSR/SNP data

*RepMiner * http://jestill.myweb.uga.edu/RepMiner.htm	Takes a graph theory approach to the identification and assembly of transposable elements from small DNA fragments resulting from subcloning bacterial artificial chromosome libraries

*SPAGeDi * http://www.ulb.ac.be/sciences/ecoevol/spagedi.html	Spatial Pattern Analysis of Genetic Diversity (SPAGeDi) is a new computer package—replacing AutocorG that was distributed to a limited extent—primarily designed to characterize the spatial genetic structure of mapped individuals and/or mapped populations using genotype data of any ploidy level

*Structure * http://pritch.bsd.uchicago.edu/structure.html	A free software package for using multilocus genotype data to investigate population structure

*TEnest * http://bak.public.iastate.edu/te_nest.html	Automated chronological annotation and visualization of maize nested transposable elements
